# Acute phase proteins and total antioxidant capacity in free-roaming cats infected by pathogenic leptospires

**DOI:** 10.1186/s12917-023-03697-y

**Published:** 2023-09-07

**Authors:** Andrea Murillo, Josep Pastor, Emmanuel Serrano, Asta Tvarijonaviciute, José Cerón, Marga Goris, Ahmed Ahmed, Rafaela Cuenca

**Affiliations:** 1 Departament de Medicina i Cirurgia Animals, Wildlife Ecology & Health group (WE&H), Facultat de Veterinària, Universitat Autònoma de Barcelona (UAB), Barcelona, 08193 Spain; 2 Departament de Medicina i Cirurgia Animals, Facultat de Veterinària, Universitat Autònoma de Barcelona, Barcelona (UAB), Barcelona, 08193 Spain; 3 Servei d’Ecopatologia de Fauna Salvatge (SEFaS), Universitat Autònoma de Barcelona (UAB), Barcelona, 08193 Spain; 4 Interdisciplinary Laboratory of Clinical Analysis Interlab-UMU, University of Murcia, Murcia, 30100 Spain; 5 OIE and National Collaborating Centre for Reference and Research on Leptospirosis (NRL), Amsterdam UMC, University of Amsterdam, Medical Microbiology, Amsterdam, 1105 AZ the Netherlands

**Keywords:** Albumin, Haptoglobin (Hp), *Leptospira spp.*, Paraoxonase-1 (PON1), Principal Component Analysis (PCA), Serum Amyloid (SAA) and Total Antioxidant Capacity (TAC)

## Abstract

**Background:**

Leptospirosis is a neglected but widespread zoonotic disease throughout the world. Most mammals are hosts of *Leptospira spp.,* including domestic cats, species in which no consensus has been reached on the clinical presentation or diagnosis of the disease. The study of acute-phase proteins (APPs) and biomarkers of oxidative status would contribute to knowledge about the disease in cats. This report evaluated four APPs: Serum amyloid A-SAA, Haptoglobin–Hp, albumin and Paraoxonase 1-PON1 and the antioxidant response through Total Antioxidant Capacity-TAC, in 32 free-roaming cats. Cats were classified as seroreactive for anti-leptospiral antibodies (group 1, *n* = 8), infected with *Leptospira spp* (group 2, *n* = 5) and leptospires-free cats (group 3, *n* = 19).

**Results:**

SAA differences were observed between groups 1 and 2 (*p*-value = 0.01) and between groups 2 and 3 (*p*-value = 0.0001). Hp concentration differences were only detected between groups 2 and 3 (*p*-value = 0.001). Albumin concentrations only differed between groups 1 and 3 (*p*-value = 0.017) and 2 and 3 (*p*-value < 0.005). Cats in groups 1 (*p*-value < 0.005) and 2 (*p*-value < 0.005) had lower PON1 concentrations than group 3. No statistically significant differences between pairs of groups were detected for TAC concentrations. The principal component analysis (PCA) retained two principal components, (PC1 and PC2), explaining 60.1% of the observed variability of the inflammatory proteins and the antioxidant TAC.

**Conclusions:**

Increases in Serum SAA, Hp, and decreases in PON1 activity may indicate an active inflammatory state in infected cats (currently or recently infected).

## Introduction

Leptospirosis is a bacterial disease, due to a spirochete bacterium of the genus *Leptospira*. It is a widespread zoonosis, which is also referred to as a neglected disease. It affects domestic and wild animals worldwide [1], mainly domestic animals. A wide variety of mammalian species, including domestic cats [2], can be susceptible to *Leptospira spp*. infection in the Americas [3–5], Asia [6, 7], Australia [8] and Europe [9, 10].

*Leptospira spp*. infection in cats has long been suspected [11], however, it was not until two decades ago that it was confirmed by microagglutination test (MAT) and PCR [11–13]. Nowadays, urinary shedding of pathogenic leptospires is well established in cats; they may also play an important role as a reservoir [13–17]. Once the animal has been infected, it suffers a period of bacteraemia of approximately 7 days, and leptospires can be detected in the blood. The main target organs in cats are the kidney and liver; the lung; brain and eyes may also be affected, especially in dogs. Infection in cats rarely causes severe organ damage as in dogs [18]. The kidney is the main organ in which leptospires replicate, resulting in the shedding of bacteria in the urine 10 days after infection. In cats, the clinical presentation of leptospirosis is uncommon and if present is usually mild [2, 4]. The wide and non-specific range of clinical signs can lead to misdiagnosis [12, 17]. Few papers have described clinical signs associated with infection in cats (e.g. renal injury) [4, 12, 16, 19], most of them being non-specific [17, 20, 21]. Asymptomatic cats can shed the bacteria via urine [7, 9, 14, 15], and unlike infected dogs, antibody titers to *Leptospira spp*. detected in subclinically infected cats are low [6]. The reason for that, remains unknown, although low antibody titers may be adequate to control infection in the species [4, 6]. Two recent studies in Malaysia and southern Chile have observed kidney tissue colonization and the urinary shedding of infectious *Leptospira spp*. respectively, from naturally infected cats [7, 14].

Ancillary tests such as inflammatory proteins and biomarkers of oxidative status could be useful in identifying the active state of the infection in cats, as their levels change rapidly in response to acute infections or inflammatory conditions [22].

Acute-phase proteins (APPs) are the most sensitive markers of inflammation [22] and their levels can not only be used as an early marker for the disease but also to assess inflammation, assist in diagnosis, disease monitoring and can help to detect subclinical infection and differentiate acute from the chronic disease [23–25]. Contrary to reports in other species, data regarding APPs levels in cats are scarce and most of the studies have been focused on the three proteins which have been shown to work as major APPs in the cat (alfa1-acid glycoprotein—AGP, serum amyloid A—SAA, and to a lesser extent, haptoglobin—Hp) [26]. Some studies highlight the potential of APPs as diagnostic markers in sick cats but also emphasize that the signalment of the cat needs also to be taken into consideration [27].

Use of APPs profiles involving at least a major protein (proteins that increase 10–100-fold during the inflammatory response), a moderate protein (increase two-tenfold), and a negative protein (fall in concentration), is highly recommended to differentiate between pathologic states [22]. In cats, SAA is a major acute protein and has been related to infectious conditions such as *Mycoplasma haemofelis* infection [28], feline infectious peritonitis [26], feline parvovirus [29], *Hepatozoon felis* and *Babesia* spp. among others [30]. It has also been studied as a prognostic factor in ill cats, whereby patients with a medium average value were more likely to survive than patients with a high average value [29, 31].

Hp is a positive moderate protein and albumin is a negative one [22–30]. Paraoxonase 1 (PON1), in addition to its antioxidant properties, destroying harmful oxidized lipids, the reason why it has been used in certain studies as a marker of oxidative status [32], has been considered a negative acute phase protein in several species and decreases have been reported during the acute phase response of inflammation in cattle [33], laboratory animals [34] horses [35], dogs [36] and also in cats [32].

Several investigations have been carried out concerning the usefulness of APPs in infectious diseases in cats, providing valuable information [23–32]. Nevertheless, unlike dogs, no work has been published relating APPs linked with *Leptospira spp.* infections in cats. In dogs, the C-reactive protein/Haptoglobin ratio (CRP/Hp) was significantly higher and albumin and total protein concentrations were significantly lower in non-surviving patients after *Leptospira interrogans* australis serogroup infection [37].

Some biomarkers of redox status have been studied in cats, as their abnormalities can help in detecting morbidity in many diseases [24, 38, 39]. The presence of reactive oxygen species (ROS) in the overload of antioxidant defence mechanisms results in oxidative stress. This may be due to a depletion of antioxidants, an exaggerated rise in ROS, or a mixture of the two [39]. As the measurement of different antioxidant molecules separately is impractical and their antioxidant effects are additive, the measurement of redox status has become a novel approach in recent years in companion animals, through total antioxidant capacity (TAC) assay [39, 40]. TAC is the sum of the activity of different antioxidants [41, 42]. Cats appear to be more susceptible to oxidative stress and damage, probably due to the presence of eight reactive and fragile sulfhydryl groups in their haemoglobin molecule and the splenic structure of the species [43, 44].

In this study, we investigated the behaviour of a panel of acute phase proteins (SAA, Hp, Albumin, and PON1) and TAC in free-roaming cats seroreactive or infected naturally by pathogenic leptospires (currently or recently infected cats). The ultimate purpose of the study was to determine whether these parameters could be used as a diagnostic aid for *Leptospira spp.* infection in cats.

## Materials and methods

### Selection of cases

Thirty-two serum samples from domestic short-haired cats that had been collected, during a previous study on *Leptospira spp.* prevalence in cats in Spain, were used in the current study [10]. Cats were part of a free-roaming cat spay program and a shelter’s animal neutering program. Blood samples were taken under anaesthesia for neutering. Sampling collection was performed under the guidelines of the Ethical Committee Animal Care and Research (CEEAH), Universitat Autònoma de Barcelona (UAB), approval number CEEAH code 2939. Written informed consent was obtained from the shelters to use the animals in the study.

All animals were tested against feline immunodeficiency and leukaemia viruses (SNAP FIV/FeLV Combo Test®). All cats had unremarkable findings upon physical examination. Serum samples were stored at -80ºC until analysis. MAT was performed by direct reading following a technique described before [45]. When a reaction was observed, it was re-checked by indirect reading. Two-fold serial dilutions of serum from 1:20 to 1:160 were tested. Any antibody titer ≥ 1:20 was considered a positive value.

Previous experimental work has shown the low titer of *Leptospira spp.* antibodies developed by cats compared to dogs [46, 47]. This has also been demonstrated when cats have been vaccinated with a four-serovar canine leptospiral vaccine [19] and in prevalence studies in domestic cats, where many of them do not exceed a 1:100 titer [6, 48, 49]. Cats might have a lower antibody response than dogs [2, 6], and a short-term immune response, with a rapid decline in titers [11, 46, 47]. On the other hand, a cat previously exposed to the bacteria may not only develop low antibody titers, but it may also develop zero antibody titers. This will depend on different factors such as the infecting serovar, if it is a serovar to which the cat is already well adapted; over time the response is described as low antibody titers or no antibody titers at all [2].

DNA extracted from cats’ biological materials (blood and urine) was tested by TaqMan real-time PCR described by Ahmed AA et al., 2020 [50].

Samples were divided into three groups: Group 1 (seroreactive *n* = 8), anti-leptospiral antibodies-positive cats detected by MAT (27 serovars, belonging to 20 serogroups and 8 species of *Leptospira*). In the present study, seroreactive cats had evidence of previous contact with *Leptospira spp.* and they are considered carriers or maintenance with falling antibody titers. Pathogenic *Leptospira spp.* DNA in urine or blood was not detected in these cats. Group 2 (infected (currently or recently) *n* = 5), PCR-positive cats to *Leptospira spp*. DNA detected by PCR in blood or urine. These cats had no antibodies against *Leptospira spp.* Group 3 (control group *n* = 19), leptospires-free cats. The absence of *Leptospira spp.* infection in the animals of this group was verified by serology (negative antibodies against *Leptospira spp.* by MAT) and PCR of blood and urine (negative leptospiral—DNA amplification).

### APPs analysis

#### SAA

Serum amyloid A concentrations were determined by a human turbidimetric immunoassay, adapted to an automated analyser (Olympus AU600). This method had been described and validated previously for use in cats [51]. Serum concentrations lower than 5 μg/mL were considered normal for cats; the limit of detection was set at 0.38 μg/mL [51].

#### Hp

Serum Haptoglobin concentrations were determined using the haemoglobin-binding method with a commercial kit (Tridelta Development Ltd., Brey, Ireland). The method was previously validated for use in cats [52]. Serum concentrations lower than 3 g/L were considered normal; the limit of detection considered was 0.0088 g/L.

#### Albumin

Serum albumin was determined using a commercially available kit (Albumin OSR 6102; Olympus Life and Material Science Europe GmbH, Irish branch, Ennis, Ireland) following the instructions of the manufacturer.

#### PON1

Serum aryl esterase activity was analyzed by measuring the hydrolysis of p-nitrophenyl acetate to p-nitrophenol, based upon inhibition of enzymatic hydrolysis of 4-nitrophenyl acetate by phenyl acetate as described previously [53] with a modification to remove the substrate from the working reagent buffer and prepare it in water as a separate starting reagent that remained colourless [54]. This modification was made because p-nitrophenyl acetate is subject to considerable spontaneous hydrolysis in the reagent buffer system originally described, which causes a grossly yellowish appearance of the reagent. The starting reagent was added to initiate the kinetic reaction. Because p-nitrophenyl acetate is insoluble in water, 63 mg of this compound was dissolved in 10 mL of methanol and stored at 2° to 8 °C. This stock solution can be kept for approximately 1 week with only a small increase in free p-nitrophenol. Afterwards, 1 mL of this solution was slowly added to 20 mL of distilled water with strong stirring agitation to prevent precipitation. The aqueous solution was freshly prepared each day.

Serum samples were each mixed with 307 μL of buffer containing 50 mM Tris and 1 mM CaCl_2_ (pH, 8.0) and then freshly made substrate containing 2.5 mM p-nitrophenyl acetate in distilled water was added. After 100 s, the reaction was monitored at 405 nm at 37 °C for 210 s in an automated biochemistry analyzer (Olympus AU600). The limit of detection was 0.3 IU/mL. Serum concentrations between 3.8 to 7.3 IU/mL were considered normal for cats [32, 52].

### Antioxidant analysis

#### TAC

Total antioxidant capacity was determined by a method previously described for humans [40] and validated for cats [32]. The method is based on the oxidation of a colourless molecule, 2,2’-azinobis(3-ethylbenzo-thiazoline-6-sulfonate) (ABTS), to a blue-green ABTS*. When the coloured ABTS* is mixed with any substance that can be oxidized, it is reduced to its original colourless ABTS form again; in contrast, the reacted substance is oxidized. The colour change is spectrophotometrically monitored. The reaction rate is calibrated with Trolox, which is widely used as a traditional standard for TAC measurement assays and the assay results are expressed in mmol Trolox equivalent/L. Serum concentrations higher than 0.35 mmol/L were considered normal; the limit of detection considered was 0.02 mmol/L.

All acute phase proteins and antioxidant analyses were performed on an automated biochemistry analyser (Olympus AU600, Olympus Diagnostic, GmbH).

### Statistical analysis

For descriptive statistics, we calculated mean, minimum, maximum, and interquartile ranges (IQR) for our set of serum biomarkers. Mean comparisons between pairs of groups (e.g., Group 1 vs Group 2), were performed using a t-test based on 1000 bootstrapped replications to compensate for the small sample size in some of the groups (e.g., Group 2). Bootstrapping was conducted following Davison and Hinkley's 1997 recommendations [55]. Values of *p*-value < 0.05 were considered significant.

In line with previous research about pathophysiology in humans [56] and animal medicine [57, 58], we performed a principal component analysis (PCA) to have a more integrative picture of the effects of *Leptospira spp*. infection on the physiological response of our cats. PCA is a dimensionality reduction technique using a linear transformation applied to multidimensional data. Derived variables from the original set, in our case the set of inflammatory markers and total antioxidant capacity, may be readily visualised in 2- or 3- principal components containing the highest observed variance. Active variables are used for creating components whereas supplementary variables are not considered for the construction of the factorial axes but are used to test statistical differences in the PCA scores. These derived variables can then be compared between supplementary categorical variables (e.g., *Leptospira spp.* infection status), through a Student’s t-test. Bootstrapping for mean comparisons was performed using the library “boot” 1.3–27 version [59], PCA analysis was performed using the libraries "car" 3–0-6 version [60] "factomineR" 2.2 version [61], "factoextra" 1.0.6 version [62] and "ggplot2" 3.2.1 version [63]. R package version 1.3–27 of the R Statistical software 4.0.5 version [64].

## Results

Table 1 summarizes the distribution of the groups of cats, by age and gender. Only one cat, included in group 1, was FIV positive; all others were negative for FIV/FeLV. Details on the distribution of the *Leptospira* serovars involved in the infected animals of group 1 seroreactive and the PCR results in group 2 infected (currently or recently) are shown in Table 2.

**Table 1:** Distribution of groups of cats according to their infection status

		**Total population** ***n*: 32**	**Group 1** ***n*: 8**	**Group 2** ***n*: 5**	**Group 3** ***n*: 19**
**Gender**	Male	21	7	2	12
	Female	11	1	3	7
**Age** **(y.o.)**	Mean	2.6	2.7	0.63	3.0
	Min–Max	0.5–12.5	0.5–7	0.5–1	0.5–12.5
	SD	3	2.43	0.21	3.46

*y.o.* years old

Group 1 = seroreactive cats detected by MAT; Group 2 = infected cats (currently or recently), detected by PCR in blood or urine; Group 3 = leptospires-free cats or control group

**Table 2:** Description of leptospiral serovars involved and PCR results by group of cats

	**Cat**	**Gender**	**Group 1** ***n*: 8**	**Group 2** ***n*: 5**	**Group 3** *n***: 19**
**Infecting** **leptospiral** **serovars**			**Serovar and titer**	**Seronegative**	**Seronegative**
	1	Female	Ballum 1:20		
	2^a^	Male	Bratislava 1:20 Cynopteri 1:20		
	3	Male	Cynopteri 1:20		
	4	Male	Cynopteri 1:20		
	5	Male	Cynopteri 1:40		
	6	Male	Ballum 1:20		
	7^a^	Male	Pomona 1:20 Proechimys 1:20		
	8	Male	Sejroe 1:20		
**PCR**	5 cats	3 Female 2 Male	Negative	1 in blood 4 in urine	Negative

Group 1 = seroreactive cats detected by MAT; Group 2 = infected cats (currently or recently infected) detected by PCR in blood or urine; Group 3 = leptospires-free cats or control group. ^a^Cat positive to more than one serovar

Serum concentrations of APPs and TAC in the different groups of the study are shown in Table 3. No concentrations lower than the limit of detection for any of the APPs and TAC studied were obtained.

**Table 3:** Serum concentrations of APPs and TAC (mean, minimum and maximum, and IQR) in cats seroreactive or infected naturally (currently or recently) by pathogenic leptospires (groups 1 and 2) and leptospires-free cats (group 3)

	**SAA** **(µg/mL)**	**Hp** **(g/L)**	**Albumin** **(g/L)**	**PON1** **(IU/mL)**	**TAC** **(mmol/L)**
Group 1 (*n* = 8) ** MEAN** ** MIN–MAX** ** IQR**	0.63	4.05	2.86	3.91	0.62
	0.10–1.60	2.49–6.3	2.31–3.3	1.88–6.02	0.47–0.79
	0.825	1.23	0.54	1.33	0.13
Group 2 (*n* = 5) ** MEAN** ** MIN–MAX** ** IQR**	46.7	4.47	2.70	3.85	0.53
	0.1–130.8	1.97–8.11	2.12–3.22	3.12–4.84	0.43–0.70
	101.9	1.79	0.52	0.95	0.1
Group 3 (*n* = 19) ** MEAN** ** MIN–MAX** ** IQR**	1.44	3.60	3.20	5.35	0.58
	0.10–6.00	1.64–4.9	2.48–4.5	3.40–7.41	0.45–0.75
	1.25	1.68	0.45	1.43	0.13

*Min *Minimum, *Max *Maximum, *IQR* Interquartile range, *SAA* Serum amyloid A, *Hp* Haptoglobin, *PON1* Paraoxonase1, *TAC *Total antioxidant capacity

Group 1 = seroreactive cats detected by MAT; Group 2 = infected cats (currently or recently) detected by PCR in blood or urine; Group 3 = leptospires-free cats or control group

### Mean comparisons between groups

There were no statistically significant differences between APPs or TAC, associated with gender *(p*-value > 0.05) or age (*p*-value > 0.05) in the control group of animals (group 3).

Significant differences in mean SAA concentrations were only observed between groups 1 (seroreactive) and 2 (infected currently or recently) (, t-test = -2.97, *p*-value = 0.01) and between groups 2 (infected currently or recently) and 3 control group respectively, *t*-test = 5.08, *p*-value = 0.0001).

Regarding Hp concentrations, statistically, significant differences were only detected between groups 2 and 3 (infected currently or recently and control group, respectively, test = 5.08, *p*-value = 0.001).

Albumin concentrations only differed significantly between groups 1 and 3 (seroreactive cats and leptospires-free cats, t-test = -2.53, *p*-value = 0.017), and between groups 2 and 3 (infected currently or recently and leptospires-free cats, t-test = -4.60, *p*-value = 9.16e-05).

Cats in groups 1 and 2 had lower PON1 concentrations than their control counterparts (t-test = -3.73, *p*-value = 8.5e-04 group 1 vs group 3, t-test = -4.5, *p*-value = 1.25e-04 group 2 vs group 3).

No statistically significant differences between pairs of groups were detected for TAC concentrations.

### Principal component analysis

**Fig. 1:**
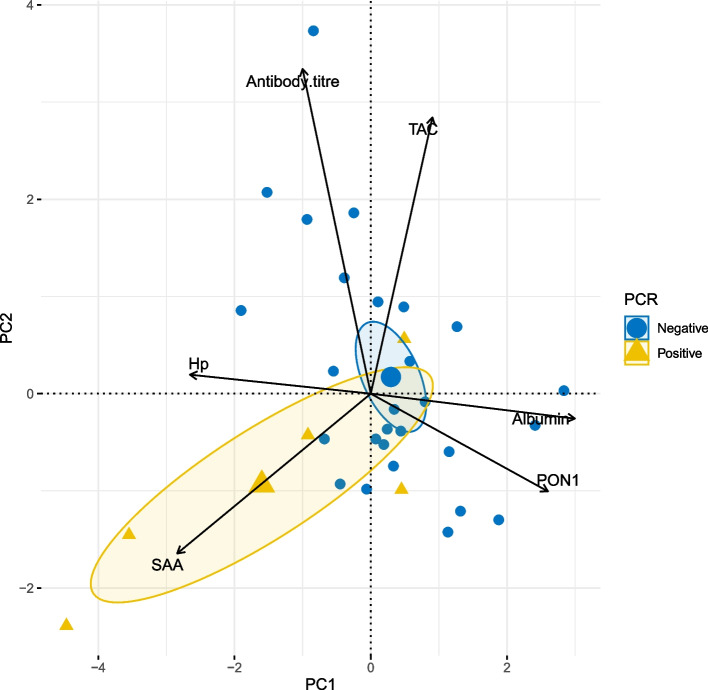
PCA biplot showing *Leptospira spp.* PCR status as a supplementary categorical variable The biplot represents the relationships between the PCR outcome of the pathogenic leptospires (positive/negative), and PC1 and PC2 components of a PCA exploring the relationships among inflammatory biomarkers (Albumin, SAA, HP, and PON1) and an antioxidant biomarker (TAC), and *Leptospira spp.* the anti-leptospiral antibody titer in 13 cats seroreactive or infected by pathogenic leptospires (currently or recently) and 19 leptospires-free cats from Spain. Neither TAC (total antioxidant capacity) nor anti-leptospiral antibodies titer contributed to the PC1

**Fig. 2:**
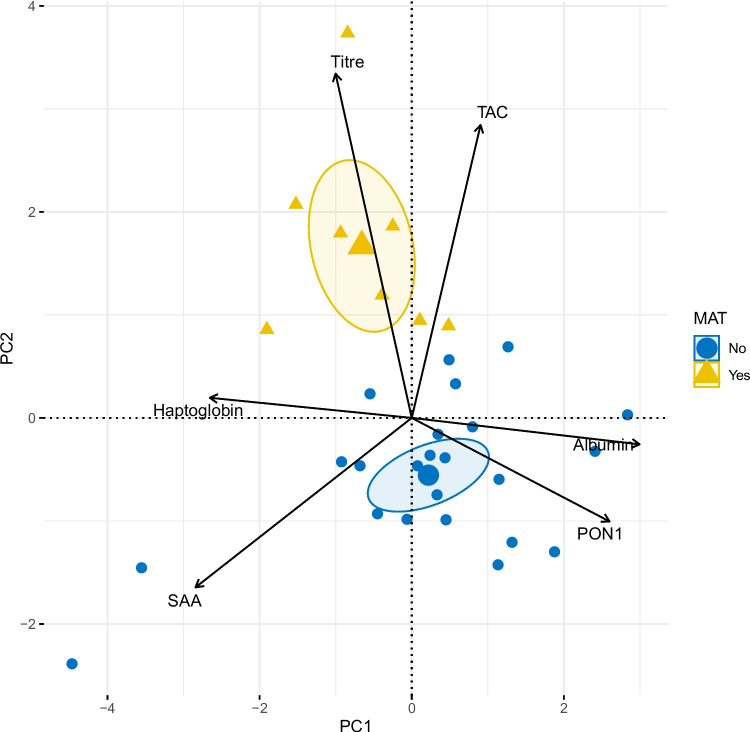
PCA biplot showing *Leptospira spp.* infection status by MAT as a supplementary categorical variable The biplot represents the relationships between MAT outcome (positive/negative), and PC1 and PC2 components of a PCA exploring the relationships among inflammatory biomarkers (Albumin, SAA, HP, and PON1), an antioxidant biomarker (TAC), and anti-leptospiral antibodies titer in 13 cats seroreactive or infected by pathogenic leptospires (currently or recently) and 19 leptospires-free cats from Spain. Inflammatory biomarkers did not contribute to PC2

The PCA revealed two principal components (PC1 and PC2), which altogether explained 60.1% of the total observed variability of the data. PC1 and PC2 explained 35.2% and 24.9% of the observed variance respectively. PC1 was mainly related to the inflammatory process and PC2 to the antioxidant response (Table 4). The main contributing variables in PC1 were albumin, SAA, HP and PON1. The presence of pathogenic *Leptospira spp*. DNA in blood or urine (positive or negative) was a complementary variable significantly related to PC1 (Fig. 1). Anti-leptospiral antibodies titer and total antioxidant capacity had the highest contributions to PC2 (Table 4). The presence of antibodies against *Leptospira spp.* by MAT (positive or negative) was a supplementary variable correlated to PC2 (Fig. 2).

**Table 4:** Contribution of active and supplementary variables to the first (PC1) and second (PC2) components of the PCA exploring the physiological response of cats seroreactive or infected naturally (currently or recently) by pathogenic leptospires

	**PC1**			**PC2**		
**Active variables**	**Contribution (%)**	**Correlation**	***p-*value**	**Contribution (%)**	**Correlation**	***p-*value**
Albumin	**27.46**	0.76	4.07e-^07^	0.28	-0.06	> 0.05
SAA	**24.71**	-0.72	2.9e-^06^	11.72	-0.42	1.7e-02
Hp	**21.56**	-0.67	2.26e-^05^	0.16	0.05	> 0.05
PON1	20.69	0.66	3.71e-^05^	4.38	-0.26	> 0.05
Anti-leptospires antibody titer	3.07	-0.25	> 0.05	**48.41**	0.85	7.64e-^10^
TAC	2.51	0.23	> 0.05	**34.05**	0.72	2.95e^−06^
**Supplementary variables**	**Contribution (%)**	**R**^**2**^	***p-*value**	**Contribution (%)**	**R**^**2**^	***p-*value**
MAT (+/-)	na	na	> 0.05	na	0.62	0.005
PCR (+/-)	na	0.22	0.006	na	> 0.05	> 0.05

na acronym indicates “not applicable”

*SAA* Serum amyloid A, *Hp* Haptoglobin, *PON1 *Paraoxonase1, *TAC *Total antioxidant capacity, MAT (+/-) = cats positive or negative to anti-leptospiral antibodies detected by MAT; PCR (+/-) = cats positive or negative *Leptospira spp.* DNA detected by PCR in blood or urine

## Discussion

We investigated the behaviour of a panel of acute phase proteins and total antioxidant capacity in free-roaming cats seroreactive or infected currently or recently, by pathogenic leptospires, to assess whether these parameters could be used as a diagnostic aid for *Leptospira spp*. infection in cats. Based on general knowledge of leptospirosis, once an animal becomes infected it may develop the incidental host state with the presentation of acute illness, which may be fatal, or a carrier state with mild or non-presenting clinical signs [2]. Acute cases of feline leptospirosis, however, are scarce [12, 17]. Nevertheless, epidemiological studies on leptospires prevalence demonstrate that the role of cats is mostly as a carrier or maintenance host. In a cat, urinary shedding of pathogenic *leptospires* has been demonstrated for 8 months [9]. Based on the above information, we believe that cats as the murine species mainly act as a carriers or maintenance instead of incidental hosts for the disease [10].

Prior studies that have noted the importance of APPs have demonstrated that markers of inflammation and oxidative stress can be associated with infectious pathologies in cats [24, 32, 65]. Research in the field has shown that APPs and TAC can be useful markers of diagnosis, follow-up, and even prognosis in the species [24, 31]. Following what was previously described [26, 66], in our study, no statistically significant differences associated with gender and age were found in the group of healthy animals, concerning APPs and TAC serum levels.

Infected cats currently or recently (group 2), showed higher levels of SAA (4.6 times higher than the upper limit (< 10 µg/mL) compared to leptospires-free cats (group 3) probably indicating an acute phase response, most likely due to the multiplication process of the bacteria in the kidneys, among other target organs, causing the release of pro-inflammatory cytokines and chemokines and the recruitment of inflammatory cells activated by the infection and tissular damage [1, 2, 18]. Similar findings have been reported by Korman et al., 2012 and Vilhena et al., 2017 in the species with other infectious diseases [28, 30].

Seroreactive animals in group 1 (anti-leptospiral antibodies-positive cats) demonstrate previous evidence of contact with the bacteria. Although scarce information is available on how long-lived are leptospiral antibodies in cats [4], it seems that anti-leptospiral antibody titer rises at the end of the first week of infection [47], the peak titer has been reported to be around day 21 [67] and in many cases, cats unlike dogs, do not develop a high antibody titer against leptospiral infection [2, 46–48]. These cats could have had a past infection, and this probably might explain the low concentration of SAA in this group. SAA is a major acute phase protein for cats and its levels increase during the acute phase of the inflammatory response, the first 24–48 h, and fall as the animal´s health status improves [65, 68]. Unfortunately, due to the cats’ origin prior history was not available.

Haptoglobin presents a high capacity for binding haemoglobin, therefore, preventing the oxidative damage that tissues can suffer due to the inherent peroxidase activity that free haemoglobin presents. Moreover, due to the capacity, it must bind to the hemo group, reducing the iron available necessary for bacterial growth [48]. Higher Hp concentrations (1.5 times higher than the upper limit (< 3 g/L) were detected in group 2 (infected currently or recently cats), in comparison group 3 (leptospires-free cats). Similar results were previously reported in infectious or inflammatory diseases in cats [24, 30, 69]. The increase of Hp concentration in the animals of group 2 supports the active infection status of the group.

No information about the role of albumin and other negative APPs in leptospiral infection in cats so far is available [70]. Albumin has been reported to decrease during feline inflammatory conditions [70, 71]. It has not been proven, however, whether this decrease depends on the extravasation of albumin from vessels to inflamed tissues or on true decreased hepatic production modulated by IL-1 that has an inhibitory effect on the synthesis of negative APPs [70]. Conversely, others reported no decrease in serum albumin levels in contrast to other APPs that exhibited changes [27]. In our study, seroreactive cats (group 1) and infected currently or recently cats (group 2) showed lower albumin concentrations compared with leptospires-free cats (group 3). Our findings support the role of albumin as a negative APP in cats seroreactive and infected (currently or recently) by pathogenic leptospires, as it has been previously reported in other inflammatory or infectious diseases in the species.

Due to the cat's origin and the lack of previous clinical history, as mentioned above, in this group, was not possible to determine characteristics related to the carrier status, i.e. intermittent shedding, length, or leptospires shedding concentration across the time [2, 9].

PON1 activity was significantly lower in cats seroreactive or infected (currently or recently) by pathogenic leptospires (groups 1 *p*-value < 0.001 and 2 *p*-value < 0.001) compared to leptospires-free cats in group 3. Given the pathophysiology of leptospiral infection, a marked inflammatory response is triggered, allowing altered levels of pro-inflammatory cytokines and probably also an acute phase protein response. In our work, the lowest activity of PON1 was obtained in naturally infected cats (currently or recently) and this fact may be because of leptospiral infection. Like other studies [32], PON1 must be considered a negative acute phase protein in domestic cats.

It should be noted that the FIV positivity of one cat in group 1, plus *Leptospira spp*. previous infection may enhance the production of inflammatory cytokines. Moreover, the low activity levels of the enzyme in both groups probably indicate higher oxidative stress compared with cats in group 3. This, however, has not been corroborated by TAC concentration in these cats as no significant changes were observed in this parameter between groups of animals (*p*-value > 0.05). Scarce information exists in the literature on the usefulness of TAC in cats and it has only been used for research purposes [38, 70].

TAC concentrations can be measured by direct or indirect assays. The direct are based on the ability to inhibit the oxidation of a substance and are made up of the Trolox assay, which is the most widely direct method used, which was employed in this study, and the oxygen radical absorbance capacity (ORAC). Indirect methods include the ferric reducing ability of plasma (FRAP) and cupric reducing antioxidant capacity (CUPRAC), which are based on the determination of the ability of a sample to reduce a metal complex. Trolox, FRAP and CUPRAC are spectrophotometric assays validated in cats [24, 32, 70], while ORAC is a fluorometric assay unvalidated in the species [42].

Some studies have analysed TAC concentration in sick cats, performing the different assays validated for the species on the same sample, the reports demonstrate that there was variation in the results according to the assay used probably because they evaluate different parameters [24, 70]. We believe that further research on TAC is required, due to differences observed when various methodologies are used for TAC determinations, therefore standardization of the technique is needed.

Considering PCA analysis, PC1 was significantly associated with the APPs profile. The negative correlation between albumin and SAA and the positive correlation between SAA and Hp are in line with previously published results [30] and confirms the decreasing blood concentrations of albumin during the inflammation response to deviate amino acid towards the synthesis of positive APPs [71]. The significance of the supplementary qualitative variable PCR (negative/positive) in the PC1 (*p-*value 0.006), suggests a relationship between the presence of the bacteria in blood or urine and the APPs responses.

PC2 was correlated with oxidative status. A positive correlation between antibody titre and TAC was described. However, the fact that seroreactive cats have low antibody titres (between 1:20 and 1:40) makes it difficult to establish a direct relationship between these two variables. *Leptospira* serovars involved in infection have also been reported to influence the inflammatory response in other species. In dogs, the Pomona serogroup [72] and Icterohaemorrhagiae [2] have been shown to trigger the strongest inflammatory responses and have the worst prognosis. In cats, this remains poorly understood [21, 46, 47, 67].

The low prevalence of the infection in domestic cats, the small sample size, and the lack of prior history and follow-up of the serum values of the biomarkers measured, due to the feral nature of the animals in the study are limitations of the present work. Despite reflecting an apparent healthy state, we cannot exclude the presence of subclinical infections other than FIV/FELV in the cats of our study.

However, to the authors’ knowledge, this is the first report evaluating serum concentrations of APPs and TAC in cats in cats with *Leptospira spp infection*. Future follow-up studies of *Leptospira ssp.* infected animals are needed to better understand the behaviour of APPs related to the oxidative status of this bacterial infection.

## Conclusion

SAA and Hp may be useful major APPs to include in the panel of laboratory tests when a leptospiral infection is suspected in cats, as their values are significantly higher in infected cats (currently or recently infected), compared to healthy non infected animals.

Seroreactive and infected naturally cats (currently or recently) by pathogenic leptospires suffer from oxidative stress, although there is a need for further studies using TAC as a measure of oxidative stress in inflammatory or infectious diseases in the species.

Patterns of inflammation, described in preliminary works, were determined, and confirmed by our PCA analysis in infected cats (currently or recently) (blood or urine).

This study provides additional information to the data available on the use of acute phase proteins, especially those considered as negative ones (albumin and PON1) and TAC, in free-roaming cats seroreactive or infected naturally (currently or recently) by pathogenic leptospires. The information could be helpful for the understanding and diagnosis of leptospirosis in cats.

## Data Availability

The datasets used and/or analysed during the current study are available from the corresponding author upon reasonable request.
